# Massive hemothorax caused by Gelpi retractor during posterior correction surgery for adolescent idiopathic scoliosis: a case report

**DOI:** 10.1186/1748-7161-9-17

**Published:** 2014-10-25

**Authors:** Long Pang, Kota Watanabe, Yoshiaki Toyama, Morio Matsumoto

**Affiliations:** Department of Orthopedic Surgery, Keio University, 35 Shinanomachi, Shinjuku, Tokyo, 160-8582 Japan; Department of Advanced Therapy for Spine and Spinal Cord Disorders, Keio University, Tokyo, Japan

## Abstract

**Background context:**

Gelpi retractors are used in surgery because they can reduce paravertebral muscle damage during retraction. No pleural injuries associated with their use in posterior spine surgery have been reported.

**Purpose:**

To describe a patient who suffered a massive postoperative hemothorax caused by a Gelpi retractor used during posterior correction surgery for adolescent idiopathic scoliosis (AIS).

**Study design:**

Case report.

**Methods:**

A case report of a rare hemothorax complication due to a Gelpi retractor is reported. The relevant literature was reviewed.

**Results:**

A 12-year-old girl with Lenke type 2 AIS, with curves of 60° at T2-7 and 75° at T7-L1, underwent posterior correction and fusion surgery using a segmental pedicle screw construct placed between T2 and L2. Although the patient’s vital signs were stable during and soon after the surgery, a chest x-ray taken one day later revealed a massive left hemothorax. Her hemoglobin concentration was decreased to 5.5g/dl, and SpO_2_ remained as low as 92% even with oxygen administration. Thoracoscopy revealed subpleural hemorrhaging at several points in the left upper intercostal area (T3-6), and a penetration of the pleura between the left 4th and 5th ribs. Active bleeding had already stopped. The tip of the Gelpi retractor appeared to have penetrated the pleura. A chest tube was placed in the patient to treat the hemothorax.

**Conclusions:**

A pleural injury by the Gelpi retractor was determined to be the cause of the hemothorax in this case. The patient’s prominent thoracic hump may have increased the risk of such an injury because the tip of a Gelpi retractor might easily have become stuck in the intercostal space rather than the paravertebral muscles.

## Background

**Figure 1:**
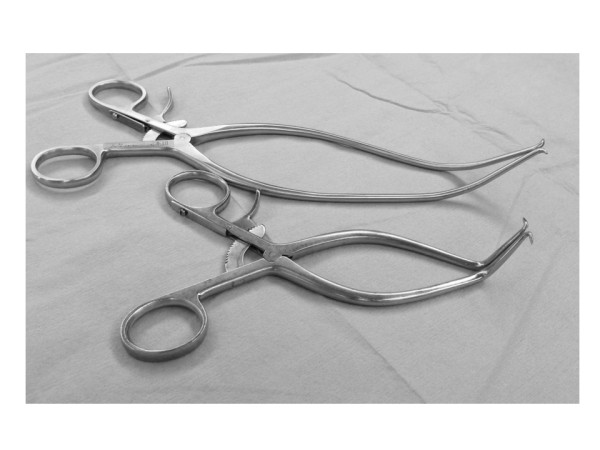
**Gelpi retractor.** The Gelpi retractor is a self-retaining retractor, which has two blades that are hinged at the middle.

Current advancements in surgical technique, spinal implants, and monitoring systems for vital organs are conducive to safe and favorable outcomes in posterior correction and fusion surgery for adolescent idiopathic scoliosis (AIS). Nonetheless, intra- and postoperative complication rates are reported to be 11.5% and 0.86% for non-neurologic and neurologic adverse events, respectively, in patients who underwent surgery [1, 2]. Of these, the reported complication rate for postoperative hemothorax is as low as 0.1% [3–5], usually associated with thoracoplasty or misplaced pedicle screws [6, 7]. Here, we describe an AIS patient who developed a hemothorax as a consequence of a pleural injury incurred by the Gelpi retractor used during surgery (Figure 1).

## Case presentation

### History and physical examination

**Figure 2:**
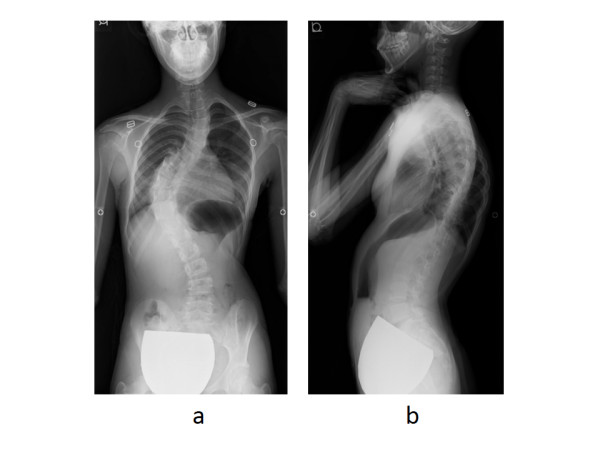
**Standing whole spine radiographs.** Standing radiographs show scoliosis of 65**°** at T2-7 and 75° at T7-12 **(a)**, and kyphosis of 61° at T5-l2 **(b)**.

A 12-year-old girl with AIS was referred to our department for surgical treatment due to progression of the curve. Physical examination revealed a prominent rib hump on the right side, and slight elevation of the left shoulder. Her body weight was 32.7 kg (-1.53SD) and height was 144.0 cm (-1.54D). Radiographs showed scoliosis of 65° at T2-7 and 75° at T7-L1, and kyphosis of 60.8° at T5-12 (Figure 2). Supine side-bending radiographs revealed scoliosis of 51.4° at T2-7 and 62.1° at T7-12, with curve flexibilities of 20.5% and 17.4%, respectively. The Lenke classification was type 2A+. Three-dimensional computed tomography (CT) images showed no congenital deformity of the thoracic or lumbar spine. Pulmonary function tests revealed a slight decrease in vital capacity (74%) and a normal forced expiratory volume in one second (86.3%).

### Surgery

The patient underwent posterior correction and fusion surgery with a pedicle screw construct between T2 and L2. After the posterior elements of the thoracic spine were exposed, pedicle screws were placed bilaterally using the ball-tip probe technique [8]. No obvious pedicle perforation was noted during screw placement. Ponte osteotomies were added at T3/4, 4/5, T7/8, 8/9, 9/10 to increase flexibility of the thoracic curves. The curves were corrected by placing a rod on the concave side of the main thoracic curve followed by a rod rotation maneuver and in-situ contouring. The scoliosis was corrected to 19° and 15°, respectively, with correction rates of 71% and 80%.

**Figure 3:**
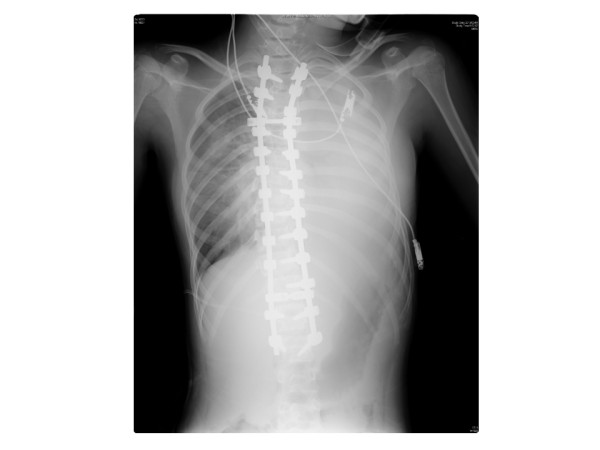
**Postoperative whole spine radiographs.** Scoliosis was corrected to 19° and 15°, with correction rates of 71% and 80% after surgery.

**Figure 4:**
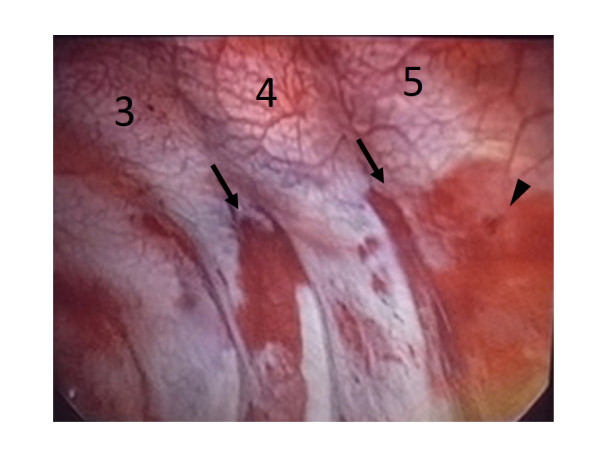
**Postoperative chest radiograph.** A chest radiograph taken 18 hours after surgery shows a massive hemothorax in the left lung.

Postoperatively, the patient’s hemodynamics were stable and the saturation of pulse oximetory (SpO_2_) was kept at 100%. Blood hemoglobin level was 8.1 mg/dl, and blood red cell count was 4.49 × 10^6^/μl. However, 18 hours later, her hemoglobin concentration had decreased to 5.5 g/dl and red cell count had fallen. SpO_2_ was 92-100% even with oxygen administration. A chest X-ray taken at that time showed a massive opacification in the left thorax (Figure 3), and a contrasted chest CT revealed a massive hemothorax on the left side. However, the CT images showed neither definitive arterial bleeding nor misplaced pedicle screws. To determine the cause of the hemothorax and treat the bleeding, thoracoscopy was performed. This analysis revealed several subpleural hemorrhages in the left T3 and T4 intercostal areas (Figure 4) and a penetration of the pleura between the left 4th and 5th intercostal areas from which active bleeding had already stopped. These complications were consistent with injuries caused by the tip of the Gelpi retractor used to expose the surgical field during surgery. A chest tube was placed to treat the hemothorax, and removed after twelve hours. The patient was discharged 10 days later without any respiratory problems.

## Discussion

The overall prevalence of non-neurologic complications associated with the surgical management of AIS ranges from 0% to 15.4% [9–13]. A study of complications after surgery on 702 AIS patients found an overall prevalence of 15.4%, with ten respiratory complications [1]. Factors including age, pulmonary function, surgical approach, number of levels fused, Lenke curve type, or region of the major curve did not correlate with an increased complication rate.

Among the perioperative complications seen in posterior correction and fusion surgery for AIS, hemothorax is quite rare, and usually associated with thoracoplasty or misplaced pedicle screws [4, 14]. Systematic reviews of 1666 and 5654 AIS patient records each identified only one instance of hemothorax following posterior surgery [3, 4]. These cases originated from thoracoplasty or misplaced pedicle screws. A rongeuer used to perform the Ponte osteotomies may also have caused some vascular damage.

**Figure 5:**
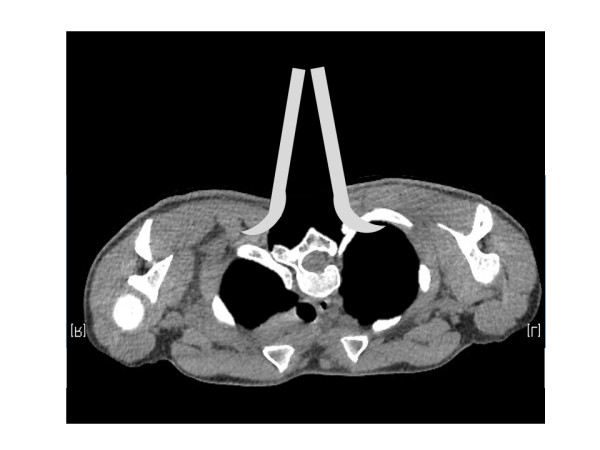
**Thoracoscopy findings.** Thoracoscopy shows subpleural hemorrhages at the left T3 and 4 ribs, and penetration of the pleura between the left 4th and 5th ribs.

The pointed tips of the blades in a Gelpi retractor push the edges of the wound apart, and retain themselves in it, easing exposure of the surgical field and reducing the intraoperative damage to paravertebral muscles during retraction (Figure 1). However, in the present case, a prominent rib hump on the left side of the upper thoracic area may have increased the risk of pleural injury by the retractor tip (Figure 5). To our knowledge, this is the first report describing a postoperative hemothorax caused by a Gelpi retractor. Surgeons should be aware of this complication when using a retractor with relatively sharp tips, especially in thoracic spine with deeper wound retraction.

## Conclusions

A 12-year-old female with adolescent idiopathic scoliosis underwent posterior correction and fusion surgery using pedicle screws. Postoperatively, her pleura was revealed to have been penetrated, with some subpleural hemorrhaging. The injury was caused by the tips of a Gelpi retractor used to reduce paravertebral muscle injury during retraction.

## Consent

Written informed consent was obtained from the parents of the patient for publication of this case report and any accompanying images. A copy of the written consent is available for review from the Editor-in-Chief of this journal.
